# CROSS-NATIONAL DIFFERENCES IN AGE AND EDUCATION GRADIENTS FOR DECLINE IN VERBAL FUNCTIONING

**DOI:** 10.1093/geroni/igae098.0169

**Published:** 2024-12-31

**Authors:** Chengming Han, Octavio Bramajo, Brian Downer

**Affiliations:** The University of Texas Medical Branch, Galveston, Texas, United States; The University of Texas Medical Branch, Galveston, Texas, United States; The University of Texas Medical Branch, Galveston, Texas, United States

## Abstract

Background. Despite the well-established associations of education and higher cognition in old age, the patterns of education gradient in different social contexts have not been adequately explored. Objective. This paper explores cross national differences in cognitive decline between adults older than 50 years in the U.S., Mexico, and China. Methods. Data were drawn from Health and Retirement Study (HRS, 2012-2020), Mexican Health and Aging Study (MHAS, 2012-2021), and China Health and Retirement Longitudinal Study (CHARLS, 2011-2020). Multilevel models were used to estimate the change in verbal function across six age groups (50-55, 56-60, 61-65, 66-70, 71-75, and 76+) and six education groups. We compared the education gradients adjusting for physical health, residence, and gender. Results. HRS respondents had the highest levels of education, whereas respondents from CHARLS had the lowest. Higher educational attainment was associated with higher verbal functioning, but the education gradient was the most pronounced in CHARLS. The slopes of verbal functioning decline were steeper in respondents aged over 60 years in MHAS and CHARLS compared to HRS. However, MHAS and CHARLS respondents in the age groups of 50-60 and 76+ showed slower cognitive decline after adjusting for education. Conclusion. Middle-aged and older adults in Mexico and China experienced faster decline in verbal functioning after age 50 than in the US. These differences can be partly explained by the lower levels of education in Mexico and China. The education gradient displayed different patterns across countries. Future studies should pinpoint heterogeneity and make policies according to different social contexts.

